# Knife wound or nosebleed—where does the blood at the crime scene come from?

**DOI:** 10.1007/s00414-023-03012-2

**Published:** 2023-05-06

**Authors:** Helen Konrad, Janina Lawniczek, Christine Bajramjan, Lisa Weber, Thomas Bajanowski, Micaela Poetsch

**Affiliations:** Institute of Legal Medicine, University Hospital Essen, Hufelandstr. 55, D-45122, Essen, Germany

**Keywords:** Nasal mucosa, Body fluid identification, DNA methylation, Epigenetic analysis

## Abstract

Secretion analysis is a useful tool in forensic genetics, since it establishes the (cellular) origin of the DNA prior in addition to the identification of the DNA donor. This information can be crucial for the construction of the crime sequence or verification of statements of people involved in the crime. For some secretions, rapid/pretests already exist (blood, semen, urine, and saliva) or can be determined via published methylation analyses or expression analyses (blood, saliva vaginal secretions, menstrual blood, and semen). To discriminate nasal secretion/blood from other secretions (like oral mucosa/saliva, blood, vaginal secretion, menstrual blood, and seminal fluid), assays based on specific methylation patterns at several CpGs were set up in this study. Out of an initial 54 different CpG markers tested, two markers showed a specific methylation value for nasal samples: N21 and N27 with a methylation mean value of 64.4% ± 17.6% and 33.2% ± 8.7%, respectively. Although identification or discrimination was not possible for all nasal samples (due to partial overlap in methylation values to other secretions), 63% and 26% of the nasal samples could be unambiguously identified and distinguished from the other secretions using the CpG marker N21 and N27, respectively. In combination with a blood pretest/rapid test, a third marker (N10) was able to detect nasal cells in 53% of samples. Moreover, the employment of this pretest increases the proportion of identifiable or discriminable nasal secretion samples using marker N27 to 68%. In summary, our CpG assays proved to be promising tools in forensic analysis for the detection of nasal cells in samples from a crime scene.

## Introduction

Short tandem repeats (STRs) are commonly used in forensic casework for the identification of victims or perpetrators as well as the analysis of family relationships [1]. In the majority of cases, STR analysis is sufficient to achieve the required results. Sometimes, however, additional information about the origin of the biological material is desired to reconstruct crime scenes and further elucidate course of events [2, 3].

So far, enzymatic or immunological methods, which are based on the presence of proteins, as well as microscopic detection methods have been routinely used for trace characterization, especially regarding blood and semen samples [4]. However, these methods can neither identify other body fluids like vaginal secretion nor can they distinguish between venous/arterial blood and menstrual blood [5].

In addition to proteins, RNA can be used to identify body fluids [4, 6]. However, ribonucleic acid is significantly less stable than DNA and easily degraded by several circumstances [7], whereas DNA is one of the most robust biological compounds that remains intact after long periods of exposure to light, heat, and humidity and still allows for genetic profiling [7, 8]. Therefore, an ideal method for identifying a type of secretion would be one that does not consume additional sample material and exploits the stability of the DNA [9]. The analysis of cell-specific, differential methylation can be used as a method of secretion analysis, since cells can be distinguished from one another by their methylation pattern [10–12].

Specific CpGs in the context of body fluid analysis have already been described in the literature for the body secretions saliva, blood, semen, menstrual blood, and vaginal secretion [13–19]. The first forensic-based study to report differentially methylated genomic loci in venous blood, saliva, semen, skin epidermis, vaginal fluid, menstrual blood, and urine was done by Frumkin et al. [20]. But the reproduction of their experiments failed in 2011 [21]. In the following years, several different assays have been developed, comprising various CpGs for the discrimination of blood, saliva, semen, and vaginal fluid [13–15], later on additionally menstrual blood [16–19]. Kader et al. provide a good review of the body fluids that could have been identified by methylation analysis so far [22].

But to the best of our knowledge, nasal discharge has never been investigated in this context. The proof of presence or absence of nose secretion or nose blood in a forensic trace can serve to confirm or refute a described crime scene scenario thus being of great help in reconstructing a crime scene scenario.

This study aims to set up methylation assays for the identification of nasal secretions based on specific CpGs to distinguish not only nasal secretion but also blood derived from nose bleeding from other fluids including venous, arterial, and menstrual blood.

## Material and methods

### Samples

The study included 182 samples of 67 adult individuals (age range 18–94 years) comprising 35 nasal mucus samples, 39 oral mucosa/saliva samples, 35 blood samples, 29 vaginal fluid samples, 21 menstruation blood samples, and 23 semen samples. No information was available about diseases or operations like vasectomy or hysterectomy. Samples were collected between 2021 and 2022 in the Institute of Legal Medicine, University Hospital Essen, Germany.

### Compliance with ethical standards

All samples were obtained after informed consent and with approval of the Medical Ethics Committee at the University of Duisburg-Essen in accordance with the Declaration of Helsinki and national laws (ethic vote number: 21–9843-BO).

### Marker

For a discrimination of nasal mucus, 27 CpGs associated to 19 different genes were chosen (Table 1) which are described in context of air pollution or air pollution-induced asthma diseases in childhood [23–26]. Additionally, 27 CpG marker regions (several CpGs per amplicon) in genes associated with formation of tight junctions were selected (Table 1) [27, 28].

**Table 1:** CpG markers, their associated genes, and their respective functions

Marker	CpG ID	Gene	Function
N_1	cg14830002	OR2B11	Signal transduction
N_2	cg23602092	TET1	Gene regulation
N_3	cg00112952	OR2B11	Signal transduction
N_4	cg20223677	DEFB104B; DEFB104A	Immune system
N_5	cg26017880	ATP9B	Energy metabolism
N_6	cg14007090	LAMA5	Extracellular matrix
N_7	cg04119977	ADCY2	Signal transduction
N_8	cg10995381	MTRR	Amino acid metabolism
N_9	cg26449294	DLG2	Signal transduction
N_10	cg09080874	DLG2	Signal transduction
N_11	cg27373604	DLG2	Signal transduction
N_12	cg08432013	DLG2	Signal transduction
N_13	cg02675969	DLG2	Signal transduction
N_14	cg05405389	DLG2	Signal transduction
N_15	cg18023263	DLG2	Signal transduction
N_16	cg14716968	DLG2	Signal transduction
N_17	cg20275558	TMEM126B; DLG2	Respiratory chain
N_18	cg06698742	TMEM126A	Respiratory chain
N_19	cg19453250	SLC39A6; ELP2	Immune system
N_20	cg16027132	chr7:105,516,844–105,517,963	
N_21	cg16518142	CDH26	Cell adhesion molecule, extracellular matrix
N_22	cg00664723	FBXL7	Signal transduction
N_23	cg24707200	NTRK1	Gene regulation, cell cycle
N_24	cg19107578	SLC9A3	Ion pump/cell metabolism
N_25	cg18749617	PCSK6	Signal transduction, transcription, etc
N_26	cg15388974	PRKD1	Signal transduction
N_27	cg20864568	MAP3K14	Signal transduction/cell proliferation
N_28	Several	JAM A	Cell adhesion molecule, extracellular matrix
N_29	Several		
N_30	Several		
N_31	Several		
N_32	Several		
N_33	Several		
N_34	Several		
N_35	Several		
N_36	Several	MUPP1	Cell adhesion molecule, extracellular matrix
N_37	Several		
N_38	Several		
N_39	Several		
N_40	Several		
N_41	Several		
N_42	Several		
N_43	Several		
N_44	Several		
N_45	Several	OCLN	Cell adhesion molecule, extracellular matrix
N_46	Several		
N_47	Several		
N_48	Several		
N_49	Several		
N_50	Several		
N_51	Several		
N_52	Several		
N_53	Several		
N_54	Several		

### DNA extraction, quantification, bisulfite conversion, amplification, and sequencing

DNA extraction was performed using DNA IQ Casework Pro Kit and Casework Extraction Kit in the Maxwell 16® instrument according to the manufacturer’s instructions (Promega, Mannheim, Germany), resulting in an extraction volume of 50 μl. DNA concentration of samples was established by real-time PCR using the PowerQuant™ System (Promega) according to the manufacturer’s instructions providing a reproducible and reliable detection threshold at least down to 25 pg DNA [29]. Using 2 μl DNA-containing solutions, each sample was analyzed in duplicates. Bisulfite conversion was performed applying MethylEdge Conversion System Kit (Promega) corresponding to the manufacturer’s instructions with an increased elution volume of 20 μl. An initial DNA amount of 50 ng was used in the conversion. DNA amplification of candidate CpGs for body fluid was done using PyroMark® PCR Kit following the manufacturer’s instructions, adapted to an increased number of 50 cycles (Qiagen, Hilden, Germany). One of the two PCR primers was biotinylated.

Sequence analysis was established in a PyroMark® Q48 Autoprep instrument using the PyroMark® Q48 Advanced CpG Reagent Kit according to the manufacturer’s instructions (Qiagen) [30]. In addition, strict attention was paid to the conditions during sequencing. For reliable results, the sequencer must be placed vibration-free and draught-free, the instrument has to be turned on at least half an hour before using, and the reagents must be at room temperature [31]. Every sample and CpG site were analyzed at least twice.

## Results and discussion

### Marker selection

In order to find nasal mucus markers, specifically regulated CpGs had to be found. Since no CpGs were mentioned in the literature in the context of body fluid identification and nasal mucus, it was decided to investigate CpGs in which methylation pattern changes have been described after NO_x_ and air pollution exposure. In all industrialized countries all over the world, people’s nasal mucosa is more or less constantly exposed to exhaust gases [24]. Therefore, changes due to this exposure could be a unique feature in the nasal mucosa leading to a distinguishable methylation pattern. Additionally, tight junctions forming cell–cell contacts are abundant in mucosa [32] so that genes involved in the forming of these characteristic features may show different methylation patterns between tissues with and without tight junctions. Genes chosen for analysis are displayed in Table 1.

### Reliability of data

Due to the demand of downstream methods, especially bisulfite conversion, all samples included in this study had a DNA concentration between 2.5 ng/μl and 50 ng/μl. For all markers, identically prepared samples with regard to extraction method or bisulfite treatment were used so that an impact of incomplete bisulfite conversion problems can be excluded.

Amplification and pyrosequencing could be successfully demonstrated for every locus included in this study. Duplicate analysis of samples showed a maximum deviation in methylation rate of 5%.

### Nasal sample identification

A DNA methylation marker that allows traces to be assigned to specific cell or tissue types should ideally show hypermethylation (> 90%) in the target and hypomethylation (< 10%) in the nontarget or vice versa [22]. To determine the suitability of the markers chosen for this study, all 54 CpG markers were analyzed in saliva, blood, and nasal secretion samples. Here, twelve of the 54 CpGs showed no amplicon after amplification (N15, N18, N25, N35, N36, N40, N41, N43, N45, N46, N49, and N54), for two of the 54 CpG markers it was not possible to design a working assay (N13 and N24), and for one of the 54 CpG markers sequencing of the desired fragment was not possible (N38). Additionally, 35 of the 54 CpG markers showed no difference in methylation percentage between saliva and nasal secretion or blood and nasal secretion. Strikingly, the associated genes of 18 of these 35 markers are often involved in signal transduction. Consequently, all 50 CpG markers mentioned above were omitted from further studies.

In the four remaining markers N2 (cg23602092), N10 (cg09080874), N21 (cg16518142), and N27 (cg20864568), DNA methylation percentage in nasal secretion varied between 11 and 26% (N2), 61% and 88% (N10), 38% and 99% (N21), and 18% and 52% (N27), respectively (Table 2). In addition to the determination of methylation levels in saliva and blood, experiments with these four markers in vaginal secretion, menstrual blood, and semen samples were conducted.

**Table 2:** Mean values and standard deviations for the selected CpG markers N2, N10, N21, and N27 and the respective numbers of analyzed samples

	Nasal secretion/blood	Oral mucosa/saliva	Blood	Vaginal secretion	Menstrual blood	Semen
	Mean value	Mean value	Mean value	Mean value	Mean value	Mean value
	Standard deviation	Standard deviation	Standard deviation	Standard deviation	Standard deviation	Standard deviation
	*n* = 35	*n* = 39	*n* = 35	*n* = 29	*n* = 21	*n* = 23
N2 (cg23602092)	17.06	29.21	30.41	16.75	5.58	6.81
	3.17	6.80	16.46	6.99	5.34	3.66
N10 (cg09080874)	74.36	88.84	70.95	79.43	75.50	95.76
	5.37	4.58	7.34	3.32	7.84	2.69
N21 (cg16518142)	64.40	92.09	92.23	81.78	78.43	98.39
	17.59	6.15	7.79	6.03	5.86	1.86
N27 (cg20864568)	33.24	20.23	25.46	15.14	23.00	90.35
	8.68	5.98	4.00	4.01	8.88	5.05

**Fig. 1:**
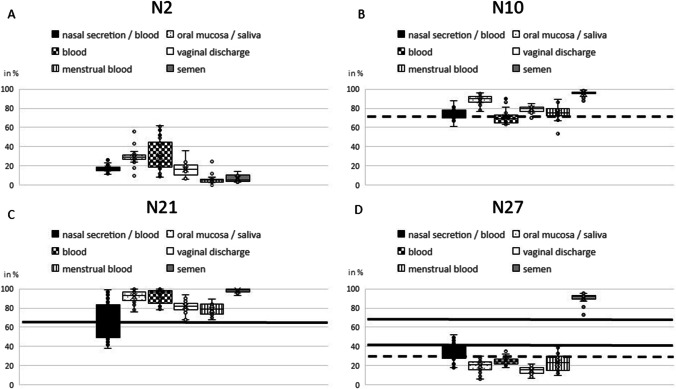
**A** N2 diagram. **B** N10 diagram. **C** N21 diagram. **D** N27 diagram; the diagrams show the different methylation levels of the various body fluids for the respective CpG marker. The solid lines define cut-off values (without pretests); the dashed lines indicate threshold values under the condition of a negative blood pretest. These two critical values, respectively, indicate the methylation range in the CpG markers in which nasal samples can be clearly identified as such

Methylation range of marker N2 demonstrated a small overlap with methylation results of blood and semen samples and a total overlap with vaginal secretion (Fig. 1A). Similarly, a small overlap of methylation results of nasal secretion to methylation range of blood samples and a total overlap to results of menstrual blood could be seen in CpG marker N10 (Fig. 1B). Therefore, no distinct cut-off value clearly discriminating nasal secretion from other body fluids could be determined for markers N2 and N10.

CpG marker N21 showed the greatest variance for methylation in nasal secretion/blood (38%–99%; mean 64%, standard deviation 18%) (Fig. 1C). Regarding saliva, blood, and semen samples, this marker demonstrated hypermethylation with mean values > 90%, whereas methylation results from vaginal secretion and menstrual blood varied between 68 and 94%. These results enabled us to set a cut-off value at 65%; every unknown sample with a N21 methylation rate lower than 65% can be clearly identified as nasal secretion/blood and discriminated from other secretions. In the samples included in this study, such an identification was possible for 22 samples out of a total of 35 analyzed nasal samples (regardless of whether they were secretions or blood) corresponding to 63% of all nasal samples.

In CpG marker N27, nasal secretions/blood showed a methylation range between 18 and 52% (mean 33%, standard deviation 9%). Overlaps to saliva, blood, vaginal secretion, and menstrual blood methylation values could be seen (Fig. 1D). Therefore, by drawing two cut-off limits > 40% and < 70%, about 26% of all tested nasal samples could be identified and discriminated from other secretions.

### Workflow for unknown samples

In unknown samples from a crime scene, it is very important to determine the sample’s origin.

Usually, starting with a blood pretest (human) which is highly specific and sensitive [33] is very useful. A positive result would confirm the presence of human blood cells, but could not distinguish between menstrual blood, nasal blood, and other sources. The application of the CpG assays N21 and N27 established in this study then determines the presence or absence of nasal blood. If no nasal epithelial cells could be found, further methylation analyses must be done to identify another source of blood cells.

A negative result of the blood test excludes the presence of nasal blood, blood, and menstrual blood. Then, our CpG assays N10, N21, and N27 could be able to identify nasal secretion if present. Here, in N21, the amount of identifiable samples does not change regardless of the pretest result. Regarding CpG marker N10, all blood negative samples with < 75% methylation include nasal cells. In this study, this allowed identification of 53% of all analyzed nasal samples (Fig. 1B dashed line). For the CpG marker N27, a negative blood pretest increases the proportion of nasal secretion samples that can be clearly differentiated from saliva, vaginal secretion, and semen by reducing the lower threshold from 40 to 30% (Fig. 1D dashed line). As a result, the percentage of clearly identifiable nasal samples could be raised from 26 to 68% in this study.

Moreover, the presence of seminal fluid could be determined directly, because its methylation values did not overlap with any other fluid in CpG marker N27. If nasal secretion and seminal fluid were excluded, a saliva pretest as well as further methylation analysis to identify vaginal secretion should be done.

**Fig. 2:**
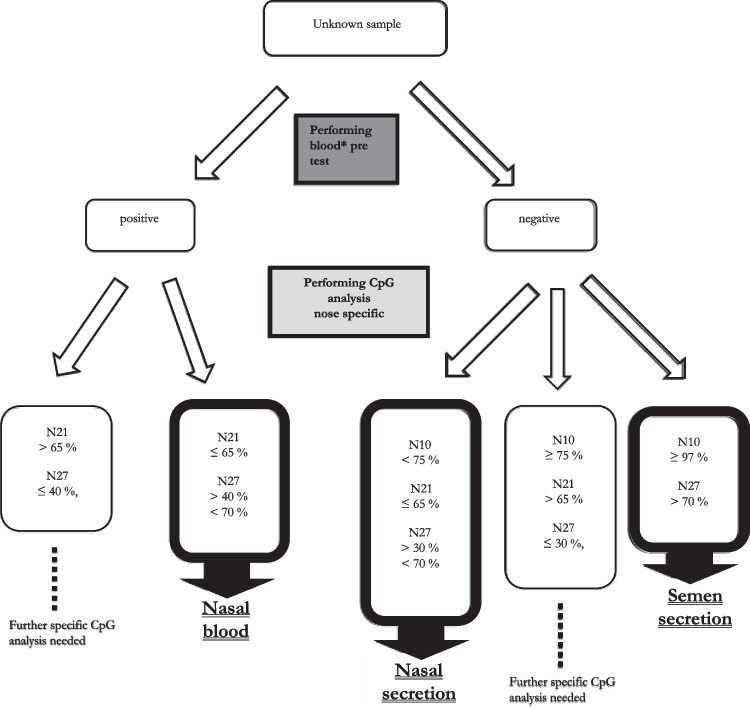
Workflow body fluid identification using pretests/rapid tests and methylation analysis; the procedure allows direct identification of nasal blood, nasal secretions, and semen. *It is useful to perform other pre/rapid tests in addition to a blood pretest

In summary, our workflow allows several outcomes in that an unknown sample may be identified directly (Fig. 2), e.g., a sample with a negative blood pretest and a methylation value of 47% in CpG marker N21 definitely identifies nasal secretion. On the other hand, an unknown sample with a positive blood pretest and a methylation value of 35% in the CpG marker N27 could still be blood from any possible source and requires further analysis.

An even greater problem is the identification of mixture samples of several body fluids [18, 34]. Since a blood pretest only detects the presence of blood cells, a positive test does not exclude the presence of cells from other sources. For example, a menstrual blood sample may also contain sperm. In order to be able to determine such mixtures as well, artificial composite samples must be created and analyzed in the next step. So further analyses (CpG marker assays and pretests) must be carried out in order to identify the composition of an unknown sample thus establishing a more complete workflow to identify and discriminate all seven body fluids and mixtures thereof in a forensic genetic context.

## Conclusion

In this study, it was possible to identify nasal mucosa-specific CpG markers and to set up methylation assays for the identification or discrimination of nasal samples. Even if an unambiguous determination of nasal secretion is not possible in 100% of samples, the results obtained so far are applicable to legally relevant questions in many cases. By optimizing or extending our workflow with additional CpG markers specific for other secretions, the unambiguously determinable proportion of unknown secretion samples can be increased.

## Data Availability

Original data are available in the institute of legal medicine Essen.
